# CFAGO: cross-fusion of network and attributes based on attention mechanism for protein function prediction

**DOI:** 10.1093/bioinformatics/btad123

**Published:** 2023-03-08

**Authors:** Zhourun Wu, Mingyue Guo, Xiaopeng Jin, Junjie Chen, Bin Liu

**Affiliations:** School of Computer Science and Technology, Harbin Institute of Technology, Shenzhen, Guangdong 518055, China; School of Electronic, Electrical and Communication Engineering, University of Chinese Academy of Sciences, Beijing 101408, China; College of Big Data and Internet, Shenzhen Technology University, Shenzhen, Guangdong 518118, China; School of Computer Science and Technology, Harbin Institute of Technology, Shenzhen, Guangdong 518055, China; School of Computer Science and Technology, Harbin Institute of Technology, Shenzhen, Guangdong 518055, China; School of Computer Science and Technology, Beijing Institute of Technology, Beijing 100081, China; Advanced Research Institute of Multidisciplinary Science, Beijing Institute of Technology, Beijing 100081, China

## Abstract

**Motivation:**

Protein function annotation is fundamental to understanding biological mechanisms. The abundant genome-scale protein–protein interaction (PPI) networks, together with other protein biological attributes, provide rich information for annotating protein functions. As PPI networks and biological attributes describe protein functions from different perspectives, it is highly challenging to cross-fuse them for protein function prediction. Recently, several methods combine the PPI networks and protein attributes via the graph neural networks (GNNs). However, GNNs may inherit or even magnify the bias caused by noisy edges in PPI networks. Besides, GNNs with stacking of many layers may cause the over-smoothing problem of node representations.

**Results:**

We develop a novel protein function prediction method, CFAGO, to integrate single-species PPI networks and protein biological attributes via a multi-head attention mechanism. CFAGO is first pre-trained with an encoder–decoder architecture to capture the universal protein representation of the two sources. It is then fine-tuned to learn more effective protein representations for protein function prediction. Benchmark experiments on human and mouse datasets show CFAGO outperforms state-of-the-art single-species network-based methods by at least 7.59%, 6.90%, 11.68% in terms of m-AUPR, M-AUPR, and Fmax, respectively, demonstrating cross-fusion by multi-head attention mechanism can greatly improve the protein function prediction. We further evaluate the quality of captured protein representations in terms of Davies Bouldin Score, whose results show that cross-fused protein representations by multi-head attention mechanism are at least 2.7% better than that of original and concatenated representations. We believe CFAGO is an effective tool for protein function prediction.

**Availability and implementation:**

The source code of CFAGO and experiments data are available at: http://bliulab.net/CFAGO/.

## 1 Introduction

Annotating protein functions is the key for unveiling the mechanism of disease, bringing great benefits for biomedical and pharmaceutical (Radivojac et al. 2013). Currently, protein functions are standardized by Gene Ontology (GO) (Ashburner et al. 2000; Carbon et al. 2021), which covers three aspects: biological process ontology (BPO), molecular function ontology (MFO), and cellular component ontology (CCO). Because biochemical experiments are expensive and time-consuming, it is impractical to experimentally annotate protein functions in large scale. In fact, only about 0.25% of known proteins have been experimentally annotated their functions (UniProt 2021). Therefore, to fill the huge vacancy of protein function annotation, developing effective and accurate computational protein function prediction methods is of great importance (Radivojac et al. 2013; Jiang et al. 2016; Zhou et al. 2019).

In the past decades, a lot of computational protein function prediction methods have been developed (Friedberg 2006; Lee et al. 2007; Rentzsch and Orengo 2009; Kihara 2016). Depending on their paradigms of feature extraction, these methods can be divided into four categories: sequence-based methods, structure-based methods, protein–protein interaction (PPI) network-based methods, and multi-source-based methods. As high sequence identity implies a similar function (Kimura and Ohta 1974; Lord et al. 2003), sequence-based methods infer protein functions by retrieving similar sequences (Cozzetto et al. 2013; Radivojac et al. 2013; Gong et al. 2016; You et al. 2018; Makrodimitris et al. 2019; Kulmanov and Hoehndorf 2020; Villegas-Morcillo et al. 2021; Kulmanov and Hoehndorf 2022). However, many proteins are similar in function, but not in sequence, so sequence-only-based methods are unable to predict functions for proteins with low sequence similarity. Protein structure determines its function, and proteins with similar structures usually share similar functions even when their sequence similarities are very low (Brenner et al. 1996; Holm and Sander 1996; Rost 1999). Therefore, structure-based methods detect the structure similarity between proteins to determine the functions of target proteins (Holm and Sander 1995; Gibrat et al. 1996; Laskowski et al. 2005). However, it is expensive to determine protein structures, and the amount of protein structure data is small. Although AlphaFold2 (Jumper et al. 2021) can predict protein structures from sequences, it has a limitation on the prediction of protein multi-chain structure that is the true structure for most proteins in living cells (Varadi et al. 2022). These facts limit the application of structure-based methods. On the other hand, as high-throughput techniques can screen PPIs in genome scale, predicting protein functions from PPI networks is desirable. PPI network-based methods assume similar functions usually shared by proteins with interaction (Sharan et al. 2007) or proteins with similar topological roles in PPI networks (Milenkovic and Przulj 2008). They predict protein functions either by label propagation among network nodes (Mostafavi et al. 2008; Mostafavi and Morris 2010) or by graph embedding of PPI network (Cho et al. 2016; Gligorijevic et al. 2018). However, high-throughput PPI data are incomplete and noisy due to the technical bias (De Las Rivas and Fontanillo 2010; Luck et al. 2020). Therefore, PPI networks alone cannot compactly describe protein functions. Protein information from multiple sources is complementary, such as PPI network and sequence (Kulmanov et al. 2018; Barot et al. 2021; You et al. 2021), in addition to subcellular location (Fan et al. 2020), text and sequence (You et al. 2018), structure and sequence (Gligorijevic et al. 2021; Lai and Xu 2022), etc. The last three Critical Assessment of Functional Annotation (CAFA) challenges have shown that the combination of different information indeed achieved the best performance on protein function prediction (Radivojac et al. 2013; Jiang et al. 2016; Zhou et al. 2019).

There are two main ways to combine protein information from multiple sources. The intuitive and simple way is concatenation, which directly concatenates the representations of multiple sources as the input of classifiers (Kulmanov et al. 2018). However, the concatenation fails to remove the effect of noise information from various sources. The widely used way is the graph neural networks (GNNs), which take the PPI network as graph and other information as node attribute features (Fan et al. 2020; You et al. 2021). But the message-passing mechanism of GNNs may inherit or even magnify the noise effect in networks (Dai and Wang 2021). Besides, GNNs with deep layers may cause the over-smoothing problem that all nodes tend to learn the same representation (Li et al. 2018; Cai and Wang 2020). Thus, it is urgent to propose a new method to integrate PPI network and other protein attributes into a more powerful representation.

In this study, we propose a new method called CFAGO to cross-fuse single-species PPI network and protein biological attributes via a multi-head attention mechanism. CFAGO contains a pre-training step and a fine-tuning step, and both of them use the multi-head attention mechanism to focus on important information. The pre-training step consists of an autoencoder, which can cross-fuse the effective information while ignoring the noise in the sources. The fine-tuning step learns more distinguishing protein representations for protein function annotation. The experimental results on human and mouse datasets show that CFAGO outperforms state-of-the-art single-species network-based protein function prediction methods, including pure PPI network-based methods and GNN-based fusion methods. Both the ablation study and protein representation visualization show the multi-head attention mechanism has an important contribution to fuse features of PPI network and other sources for single-species protein function prediction.

## 2 Materials and methods

### 2.1 Datasets

We conduct experiments on two species: *Homo sapiens* (human) and *Mus musculus* (mouse). The PPI data and protein sequence data are retrieved from the STRING (v11.5) database (Szklarczyk et al. 2021). In particular, we use the ‘combined’ type PPI data, which includes all of the ‘experimental’, ‘coexpression’, ‘coocurrence’, ‘neighborhood’, ‘fusion’, ‘database’, and ‘textmining’ types of PPI data. The protein function annotation data are retrieved from Gene Ontology Resource (http://geneontology.org) (version 2022-01-13 release) (Ashburner et al. 2000; Carbon et al. 2021). Protein subcellular location and protein domain data are retrieved from the UniProt database (v3.5.175) (UniProt 2021). Specifically, the protein domain from the pfam database (Mistry et al. 2021) is used.

Following the standard CAFA protocol (Radivojac et al. 2013; Jiang et al. 2016; Zhou et al. 2019), we extract experimental annotations of protein functions with evidence ‘IDA’, ‘IPI’, ‘EXP’, ‘IGI’, ‘IMP’, ‘IEP’, ‘IC’, or ‘TA’, and use two time points: $t_{0}$ (1 January 2018), $t_{1}$ (31 December 2020), to divide annotated proteins into training, validation, and testing sets. Concretely, the training set consists of proteins that have been annotated no later than $t_{0}$, validation set consists of proteins that only have been annotated in $\left(t_{0},t_{1}\right]$, testing set consists of proteins that only have been annotated after $t_{1}$. We only use GO terms that have at least 10, 5, and 1 training, validation, and testing proteins, respectively. Furthermore, to reduce the effect of the dependence relationship between GO terms, we remove those GO terms annotating more than 5% of the species’ PPI network proteins, following a previous study (Barot et al. 2021). The statistics of GO terms, training, validation, and testing sets used in this study is shown in Table 1.

**Table 1.** Data statistics considered for each organism and Gene Ontology branch

Species	Statistics	BPO	MFO	CCO
Human	#GO terms	45	38	35
	#training proteins	3197	2747	5263
	#validation proteins	304	503	577
	#testing proteins	182	719	119
Mouse	#GO terms	42	17	37
	#training proteins	2714	1185	4014
	#validation proteins	336	232	694
	#testing proteins	155	126	147

### 2.2 Method

**Figure 1:**
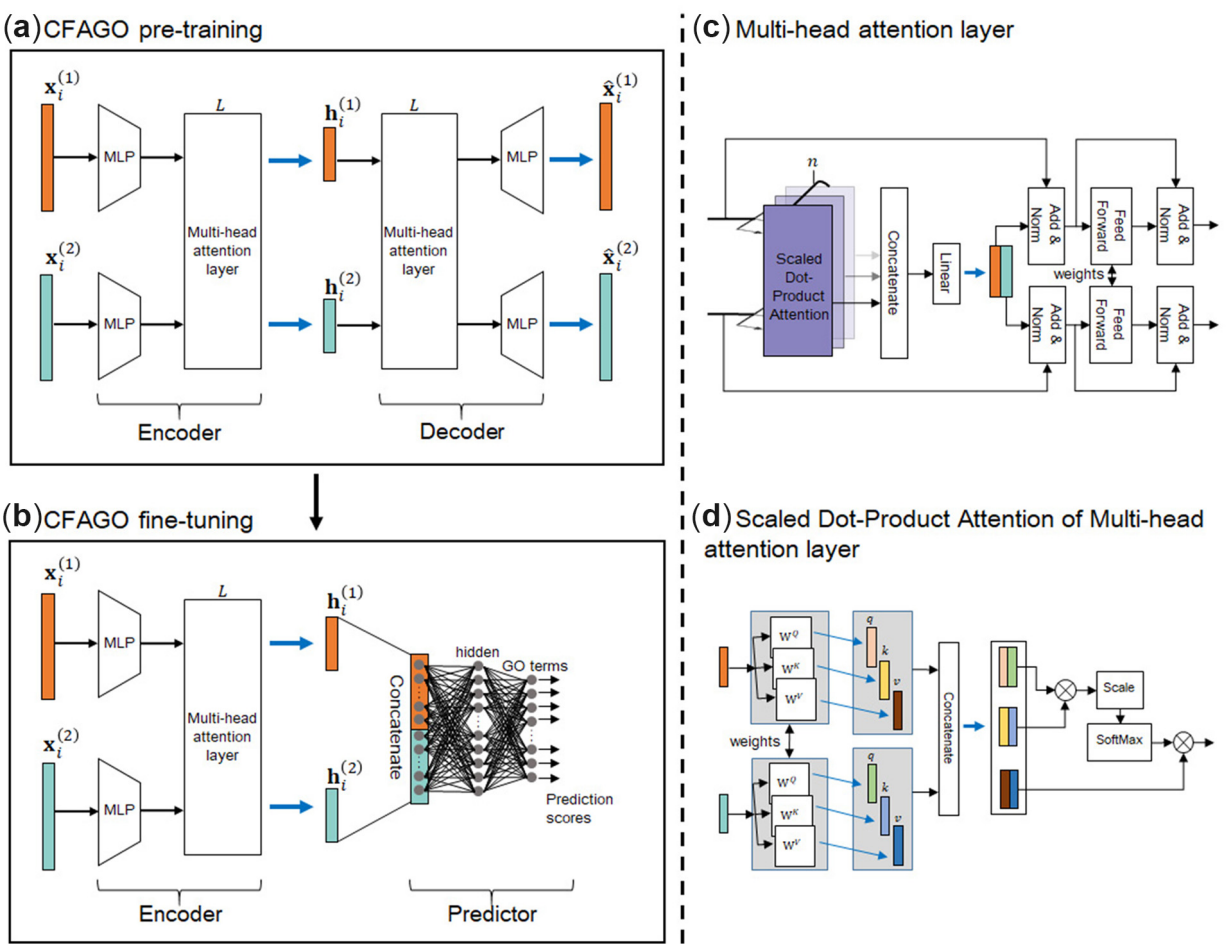
The flowchart of CFAGO. (a) Architecture of encoder–decoder for CFAGO pre-training step, where MLP stands for multilayer perceptron. (b) Architecture of CFAGO fine-tuning step. (c) Multi-head attention layer of Encoder and Decoder. (d) Scaled Dot-Product Attention of Multi-head attention layer.

CFAGO introduces multi-head attention layers to cross-fuse protein information from different sources in two steps (Fig. 1). The first step is the pre-training, which is an encoder–decoder model that learns protein hidden embedding vectors by reconstructing original source features. The second step is fine-tuning, which combines the pre-trained encoder with a two-layer fully-connected neural network to predict protein functions.

#### 2.2.1 PPI network structure and node attribute representations

For a protein, we use its first-order neighborhood of the PPI network to represent its network structure. Specifically, we first convert the PPI network into a weighted adjacency matrix, in which elements are weights of edges, then normalize elements to range $\left[0,1\right]$ by min-max normalization. A column vector of the normalized adjacency matrix is a protein representation that contains normalized weights to its first-order neighborhoods.

For the protein attributes, we select the widely used protein domain and subcellular location information. Protein attributes are represented as binary vectors by bag-of-words encoding, which assigns 1 to an element in the binary vector if the protein is annotated with the corresponding domain or subcellular location. We filter out protein domain terms that appear less than 6 times in the dataset, following a previous study (Fan et al. 2020). Without prior knowledge of different attributes and GO aspects, we concatenate the two attribute vectors as the protein attribute vector representation for all GO aspects.

#### 2.2.2 Multi-head attention layer

Here we define the multi-head attention layer following a previous study (Vaswani et al. 2017). The multi-head attention layer consists of multi-head attention, residual connection, normalization, and position-wise feed-forward networks. The core of multi-head attention is the Scaled Dot-Product Attention (Vaswani et al. 2017): where Q,K,V are the query matrix, key matrix, and value matrix, respectively, and $d_{k}$ is the dimension size of key matrix. The multi-head attention is defined as (Vaswani et al. 2017): where n is the number of heads, ${\mathrm{head}}_{j}$ is defined as: where $W_{j}^{Q}\in R^{d\times d_{k}}$, $W_{j}^{K}\in R^{d\times d_{k}}$, $W_{j}^{V}\in R^{d\times d_{k}}$, $W^{O}\in R^{d\times d_{k}}$ are projection weight parameter matrices. Here d is the dimension size of hidden embedding vectors, and $d_{k}=d/n$.


(1) $$\mathrm{Attention}\left(Q,K,V\right)=\mathrm{softmax}\left(\frac{QK^{T}}{\sqrt{d_{k}}}\right)V$$

(2) $$\mathrm{MultiHead}\left(Q,K,V\right)=\mathrm{Concat}\left({\mathrm{head}}_{1},\ldots {\mathrm{head}}_{n}\right)W^{O}$$

(3) $${\mathrm{head}}_{j}=\mathrm{Attention}\left(QW_{j}^{Q},KW_{j}^{K},VW_{j}^{V}\right)$$

The feed-forward network consists of two fully connected layers with a nonlinear activation function (Vaswani et al. 2017): where f is the nonlinear activation function, $W_{1}\in R^{d\times d_{ff}}$, $W_{2}\in R^{d_{ff}\times d}$ are the weight parameter matrices of feed-forward network, $d_{ff}$ is the output dimension size of the first linear layer, $b_{1}\in R^{d_{ff}}$, $b_{2}\in R^{d}$ are bias parameter vectors.


(4) $$\mathrm{FFN}\left(h\right)=W_{2}f\left(W_{1}h+b_{1}\right)+b_{2}$$

#### 2.2.3 Pre-training with a self-supervised encoder–decoder

The pre-training step uses an encoder–decoder model to cross-fuse information from two sources. For protein i, its two original source features are represented as $x_{i}^{\left(1\right)}\in R^{d\left(1\right)}$ and $x_{i}^{\left(2\right)}\in R^{d\left(2\right)}$, where $d\left(m\right)$ is the feature dimension of source m.

**Encoder:** the encoder has two parallel multilayer perceptrons (MLPs), each for a source feature, and *L* multi-head attention layers. As original features of different sources may be sparse and differ in dimension, the original feature vector of protein i from source m, $x_{i}^{\left(m\right)}$, is projected to a common vector with d dimensions by a two-layer MLP, which is defined as: where f is the nonlinear activation function, LN is the layer normalization function (Ba et al. 2016), $W_{1}\in R^{d(m)\times d_{e}}$ and $W_{2}\in R^{d_{e}\times d}$ are the weight matrices, $b_{1}\in R^{d_{e}}$and $b_{2}\in R^{d}$ are the bias vectors, $d_{e}$ is the size of the MLP hidden layer. Then, the projected vectors of two sources are cross-fused by multi-head attention layers to generate protein-hidden embedding vectors.


(5) $$\mathrm{MLP}\left(x\right)=f\left(\mathrm{LN}\left(W_{2}f\left(\mathrm{LN}\left(W_{1}x+b_{1}\right)\right)\right)+b_{2}\right)$$

**Decoder:** the structure of the decoder is symmetric to the encoder. The decoder first feeds the hidden embedding vectors into *L* multi-head attention layers. Then for protein *i*, the feature vector of source m is reconstructed by an MLP whose structure is symmetric to the corresponding MLP in encoder, denoting as $\;{\hat{x}}_{i}^{\left(m\right)}$. In decoder, the sigmoid function is used as the activation function of the output layer of MLPs.

The aim of the encoder–decoder is to minimize the sample-wise binary cross-entropy loss between original and reconstructed source features: where N is the number of total proteins in PPI network, $x_{ij}^{\left(m\right)}$ and ${\hat{x}}_{ij}^{\left(m\right)}$ are the *j*th dimension value of $x_{i}^{\left(m\right)}$ and ${\hat{x}}_{i}^{\left(m\right)}$, respectively, Θ is the set of all parameters in the pre-training step.


(6) $$\mathrm{loss}\left(\Theta \right)=\frac{1}{N}\sum_{i=1}^{N}\sum_{m=1}^{2}\sum_{j=1}^{d\left(m\right)}-\left[x_{ij}^{\left(m\right)}\log\;{\hat{x}}_{ij}^{\left(m\right)}+\left(1-x_{ij}^{\left(m\right)}\right)\log\left(1-{\hat{x}}_{ij}^{\left(m\right)}\right)\right]$$

#### 2.2.4 Fine-tuning for protein function prediction

In this study, protein function prediction is modeled as a multi-label task. We extract the pre-trained encoder and attach it with a predictor, which is a two-layer perceptron, to predict protein labels. Let the number of target GO terms to be K. The predictor takes the concatenation of the embedding vectors, denoting as $h_{i}^{\left(1\right)}$ and $h_{i}^{\left(2\right)}$, of the two sources generated by the encoder as input, and output a *K*-dimension score vector for GO terms Formally, the prediction score vector ${\left[p_{i1},\ldots p_{ik}\right]}^{T}$ of protein *i* is defined as: where || is concatenation operator, and σ is the sigmoid function, $d_{h}$ is the size of the predictor’s hidden layer, $W_{h}\in R^{2d\times d_{h}}$ and $W_{o}\in R^{d_{h}\times K}$ are the weight matrices of predictor’s hidden and output layers, respectively. $b_{h}\in R^{d_{h}}$ and $b_{o}\in R^{K}$ are the bias vectors of predictor’s hidden and output layers, respectively.


(7) $${\left[p_{i1},\ldots p_{iK}\right]}^{T}=\sigma \left(W_{o}\sigma \left(W_{h}\left(\left\|h_{i}^{\left(m\right)}\right.\right)+b_{h}\right)+b_{o}\right)$$

For GO terms, negative proteins are much more than positive proteins in training set. Therefore, we use the asymmetric loss (Ridnik et al. 2021) as the prediction loss: where $y_{ik}\in \left\{0,1\right\}$ and $p_{ik}\in \left[0,1\right]$ are the true label and predicted score of protein i in terms of GO term k, $\gamma_{+}$, and $\gamma_{-}$ are the positive and negative focusing parameters, respectively, $N_{\mathrm{train}}$ is the number of proteins in training set, Φ is the set of all parameters in the fine-tuning step. In this study we set $\gamma_{+}=0$ and $\gamma_{-}=2$.


(8) $$\mathrm{ASL}\left(\Phi \right)=\frac{1}{N_{\mathrm{train}}K}\sum_{i=1}^{N_{\mathrm{train}}}\sum_{k=1}^{K}-y_{ik}{\left(1-p_{ik}\right)}^{\gamma_{+}}\log\left(p_{ik}\right)-\left(1-y_{ik}\right){\left(p_{ik}\right)}^{\gamma_{-}}\log\left(1-p_{ik}\right)$$

### 2.3 Evaluation metrics

In this study, we use five metrics to evaluate prediction performance, including two types of area under the precision–recall curve (AUPR), e.g. micro-averaged AUPR (m-AUPR) and Macro-averaged AUPR (M-AUPR), F1-score (F1), accuracy (ACC), and F-max score (Fmax). The first three metrics are function-centric measures that evaluate proteins annotated to each GO term, while the last two metrics are protein-centric measures that evaluate GO terms annotated to each protein.The m-AUPR, M-AUPR, F1, and ACC are widely used to evaluate protein function prediction (Mostafavi et al. 2008; Cho et al. 2016; Gligorijevic et al. 2018; Barot et al. 2021). Specifically, m-AUPR is the AUPR across the vectorized results of true label and prediction matrices, M-AUPR is the average of AUPRs of all GO terms. F1 is computed by taking the top three prediction scores for each protein, then constructing a two-by-two confusion matrix for each GO term, and calculating the harmonic mean of precision and recall on the summed-up confusion matrix of all GO terms. Accuracy is the proportion of proteins that the predicted GO terms are exactly the same as the true GO terms, using 0.5 as the predicted threshold. Fmax is used for the CAFA challenging (Radivojac et al. 2013; Jiang et al. 2016; Zhou et al. 2019) and many protein function prediction studies (Kulmanov and Hoehndorf 2020; Barot et al. 2021; Gligorijevic et al. 2021; You et al. 2021; Lai and Xu 2022), which is defined as following: where τ is the threshold value, $pr\left(\tau \right)$ and $rc\left(\tau \right)$ are the precision and recall in terms of τ, respectively, which are defined as: where $I\left(\cdot \right)$ is the indicator function, $q\left(\tau \right)$ is the number of proteins whose max predicted score is not less than τ, g is the number of target proteins.


(9) $$\mathrm{Fmax}=\underset{\tau }{\max}\left\{\frac{2\times pr\left(\tau \right)\times rc\left(\tau \right)}{pr\left(\tau \right)+rc\left(\tau \right)}\right\}$$

(10) prτ=1qτ∑i=1qτ∑kIpik≥τ∧yik≡1∑kIpik≥τrcτ=1g∑i=1g∑kIpik≥τ∧yik≡1∑kIyik≡1

In addition, we use the Davies Bouldin Score (Davies and Bouldin 1979) to evaluate the goodness of feature representations. Lower Davies Bouldin Score means proteins with same functions are clustered together better.

## 3 Experiments

### 3.1 Experimental setup

We evaluate CFAGO on the three GO aspects: BPO, MFO, and CCO separately. We use the same empirical hyperparameter set for both species and all of the three GO aspects, and merge the validation and training sets for each aspect to train our model. Specifically, the batch size is 32 for both of pre-training and fine-tuning steps, the encoder MLP hidden dimension size $d_{e}=1024$, hidden embedding vector dimension size d=512, the number of multi-head attention layers L=6, feed-forward network hidden dimension $d_{ff}=2048$, number of attention heads n=8, predictor hidden layer dimension $d_{h}=256$, normalization function is set as the layer normalization (Ba et al. 2016). We use the Gaussian Error Linear Unit (Hendrycks and Gimpel 2016) as the nonlinear activation in all hidden layers in encoder and decoder, followed by a dropout layer. In predictor, the dropout rate is set as 0.3, while in encoder and decoder, the dropout rate is set as 0.1. The pre-training step is trained with 5000 epochs, learning rate of 1e-5 for the first 2500 epochs and 1e-6 for the remaining epochs. The fine-tuning step is trained with 100 epochs. In the first 50 epochs, we freeze the pre-trained encoder, and set the learning rate of 1e-4 for predictor. In the last 50 epochs, we set the learning rate of 1e-6 for the encoder, and set the learning rate of 1e-5 for predictor. The optimizer is AdamW (Loshchilov and Hutter 2019).

Here, we compare CFAGO with eight state-of-the-art PPI network-based methods, including two baseline methods, four network integrate methods, and two GNN-based methods. The baseline methods include the Naïve and BLAST methods of CAFA (Radivojac et al. 2013). The network integrate methods include deepNF (Gligorijevic et al. 2018), Mashup (Cho et al. 2016), GeneMANIA (Mostafavi et al. 2008), and NetQuilt (Barot et al. 2021). The deepNF, Mashup, and GeneMANIA integrate multiple types of single-species PPI networks into a single kernel or compact low-dimensional representations, while NetQuilt globally aligns different species’ PPI networks into a meta-network profile. The GNN-based methods include Graph2GO (Fan et al. 2020) and DeepGraphGO (You et al. 2021). Since this study focuses on the information cross-fusion from multi-sources of single species, all of the competing methods are fitted on single species datasets by hyperparameters reported on their papers. Besides, their features are generated by using their feature-generating tools or procedures. Because GeneMANIA, Mashup, deepNF, and Graph2GO did not conduct experiments on mouse, we use their reported structure and hyperparameters for human to evaluate their performance on both species. For Graph2GO, the validation and training sets of each aspect are merged to train the model. All of the results are averaged by five random repeats.

### 3.2 CFAGO outperforms competing methods

**Figure 2:**
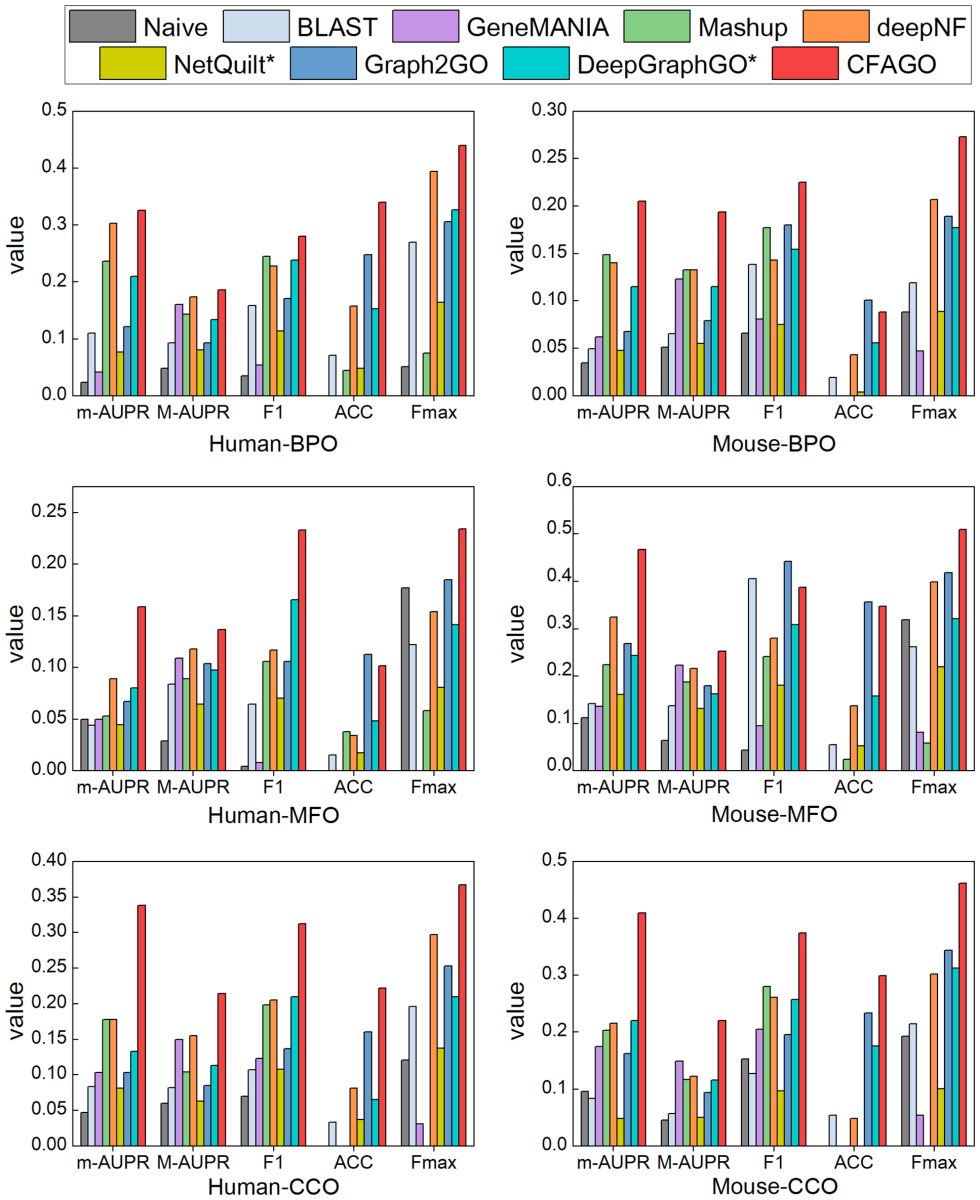
Performance comparison of CFAGO with competing methods. CFAGO achieves better or comparable performance compared to the competing methods in terms of all measurements. The blank gaps on the ACC and Fmax measurements mean the value of corresponding methods are 0. Methods labelled with an asterisk are multi-species methods but have been trained as single-species methods in the comparisons.

Figure 2 shows the performance of different methods on testing datasets of human and mouse for the three GO aspects. It is clear that CFAGO outperforms all of the competing methods in terms of m-AUPR, M-AUPR, and Fmax measures. Specifically, in terms of m-AUPR, CFAGO achieves (7.59%, 37.58%), (78.65%, 43.69%), and (89.89%, 85.91%) higher than that of competing methods on (human, mouse) datasets for BPO, MFO, and CCO, respectively. CFAGO achieves (6.90%, 45.86%), (16.10%, 13.45%), and (38.01%, 47.65%) higher in terms of M-AUPR, and (11.68%, 31.88%), (26.47%, 22.01%), and (23.57%, 34.30%) higher in terms of Fmax than that of competing methods on (human, mouse) datasets for BPO, MFO, and CCO, respectively.

For the F1 measurement, CFAGO also outperforms the competing methods, only except on mouse MFO where BLAST and Graph2GO achieve better performance. This is not surprising, because several studies have pointed out that MFO is highly correlated with sequence information (Lord et al. 2003; Fan et al. 2020), and Graph2GO has the additional information from protein sequence similarity networks, in addition to PPI networks. For the ACC measurement, CFAGO outperforms competing methods on half of tasks. For the worst case of CFAGO, it has comparable performance with Graph2GO on human MFO, mouse BPO, and MFO, while outperforming other seven competing methods. We also noticed that the frequencies of individual labels on all datasets except on mouse BPO are much lower than 0.5 in the training dataset. Since Naïve uses the frequency of each label on the training dataset as the prediction score for every test protein, it achieves a value of 0 in terms of the ACC measurement on the corresponding datasets. Besides, as training proteins cover less than 30% of total proteins in the PPI network, GeneMANIA cannot effectively propagate labels from training proteins to test proteins, and Mashup is unable to learn protein compact low-dimensional via matrix factorization, leading them achieve a value of 0 in terms of ACC and Fmax on several tasks.

We further investigate the AUPR performance of CFAGO in individual GO terms. The results in Supplementary Figs S1–S6 show that the performance of linked GO terms is not correlated. These results are expected. The first reason is that proteins are annotated to the most granular term (Ashburner et al. 2000), the second reason is that we remove those annotating more than 5% of proteins in the PPI network to reduce the effect of the dependence relationship between GO terms.

Such outstanding results of CFAGO demonstrate the feature cross-fusion via multi-head attention mechanism has obvious advantages compared with pure PPI network-based methods and the multi-source combining method based on GNN.

### 3.3 Attention mechanism learns more distinguishing representation via cross-fusing information from multiple sources

To quantitatively evaluate the effectiveness of cross-fusion by attention mechanism and pre-training, we compare the distinguishing power of protein representations by Davies Bouldin Score (Davies and Bouldin 1979).

We compare four types of protein representations, including the original PPI network representation, original attribute representation, and hidden embedding representations learned by CFAGO with and without attention mechanism. The structure of CFAGO without multi-head attention mechanism is shown in Supplementary Fig. S7. The GO term sets are used as the protein cluster labels, that is, two proteins with exactly the same GO term set are considered to be in the same cluster. The results on the union of training and validation set are listed in Table 2, which shows that the hidden embedding representations learned by full CFAGO model achieve the best performance. Specifically, its Davies Bouldin Score is (6.15%, 8.39%), (8.02%, 5.51%), and (6.30%, 7.63%) lower than that of comparison representations on (human, mouse) datasets for BPO, MFO, and CCO, respectively. These results indicate that cross-fusion of CFAGO can effectively fuse protein features from multiple sources to generate better protein representation for function prediction.

**Table 2:** Davies Bouldin Score comparison of different protein feature represents

Representation	Human			Mouse		
	BPO	MFO	CCO	BPO	MFO	CCO
o_PPI^a^	1.855	2.250	2.243	1.991	3.133	2.204
o_attribute^b^	2.128	2.387	2.128	2.209	3.349	2.122
c_embedding^c^	1.884	2.183	2.201	1.943	2.924	2.179
cf_embedding^d^	**1.741**	**2.008**	**1.994**	**1.780**	**2.763**	**1.960**

*Note:* Smaller Davies Bouldin Score value means the cluster of protein representations are more clearly separated.

a The original PPI network structure feature.

b The original attribute feature.

c The concatenation of hidden embedding vectors output by CFAGO without attention mechanism.

d The concatenation of hidden embedding vectors output by CFAGO.

**Figure 3:**
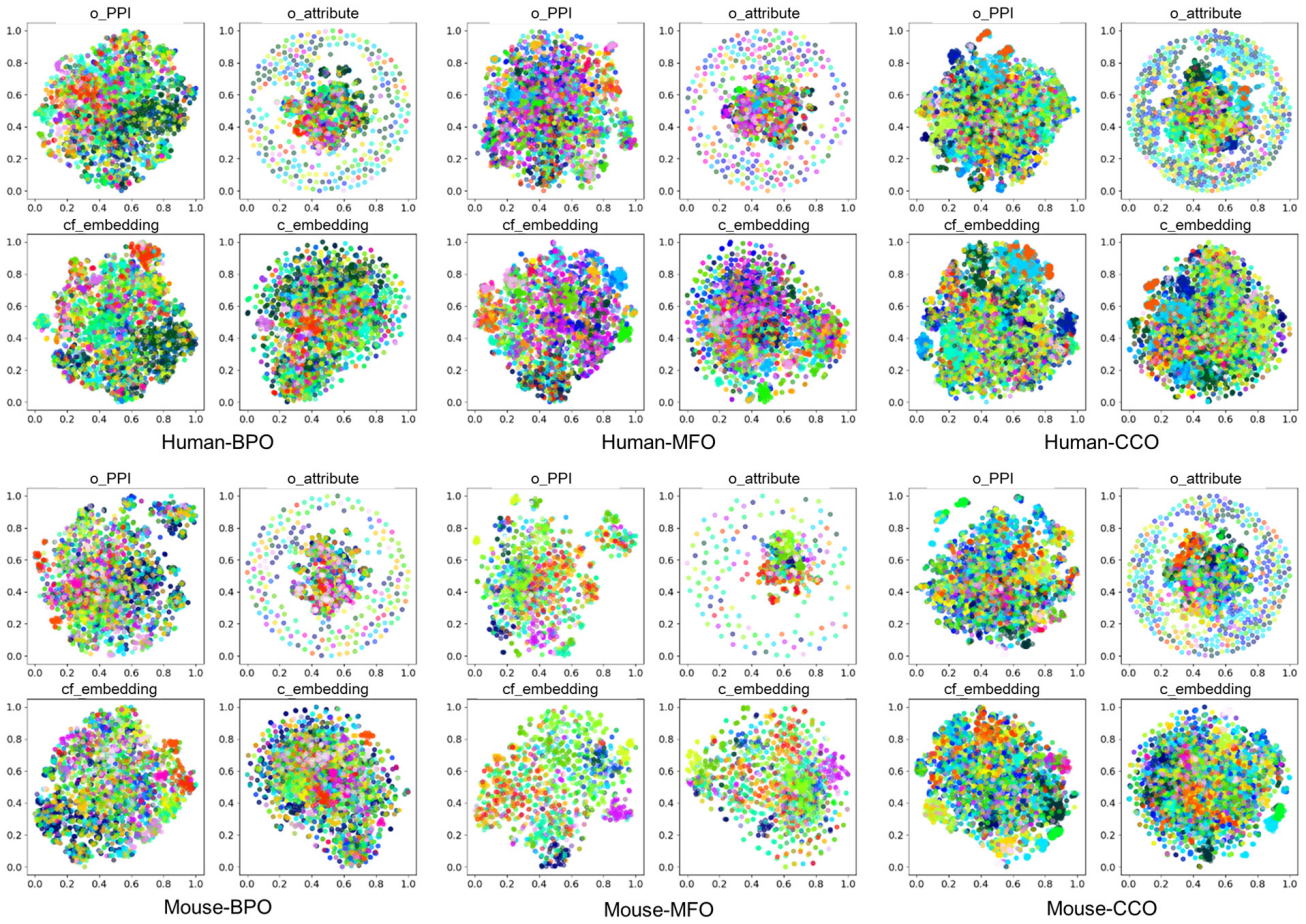
Visualization of different feature representations on human and mouse dataset in terms of the BPO, MFO, and CCO, respectively. o_PPI is the original PPI network structure feature, o_attribute is the original biological attribute feature, cf_embedding and c_embedding is the concatenation of hidden embedding vectors output by CFAGO, with and without attention mechanism, respectively.

In addition, we visualize the distribution of above protein representations on human and mouse datasets for the three aspects of GO via t-Distributed Stochastic Neighbor Embedding (van der Maaten and Hinton 2008) by assigning a unique color for each cluster label (GO term set), as shown in Fig. 3. It is clear that the distribution of original PPI network structure and attribute features are different, indicating they are complementary for annotating protein function. The original PPI network structure feature shows a relatively clear distribution, while the original attribute feature isolates proteins into a ring part and a nucleus part that each of which shows more vague cluster segmentation.

The representation learned by CFAGO without cross-fusion mixes up the shape of distribution of original PPI network structure and original attribute, showing an edge that is similar to the ring part in the distribution of original attribute. The hidden embedding representation learned by full CFAGO model shows a much better cluster segmentation than compared representations. These results show that the features from two sources are indeed cross-fused by multi-head attention mechanism.

### 3.4 The contribution of self-supervised pre-training and multi-head attention mechanism

**Figure 4:**
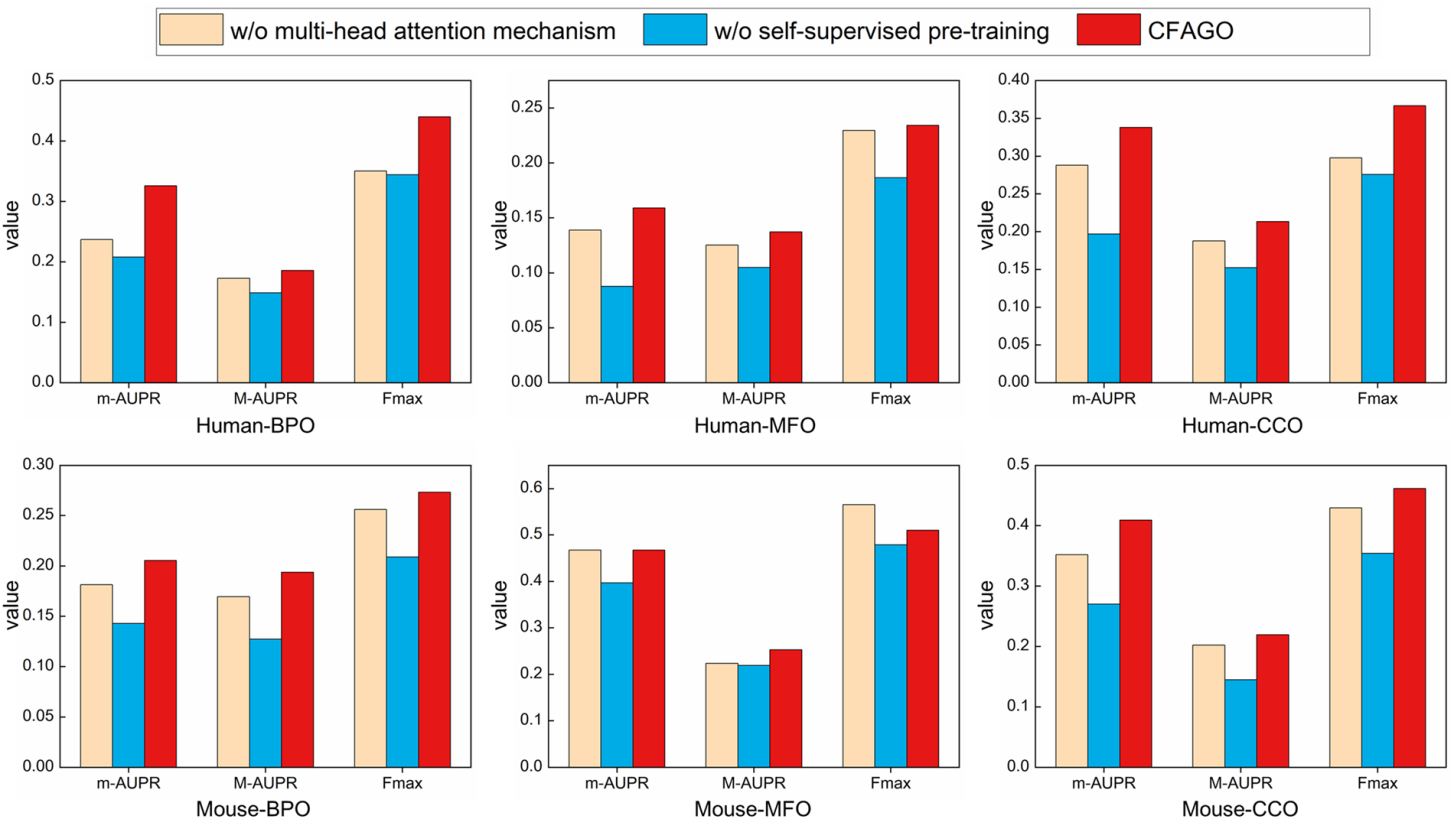
Ablation studies of self-supervised pre-training and multi-head attention mechanism, the meaning of different colors is shown on top of the graph. m-AUPR, M-AUPR, and Fmax are performance measurements.

Here we conduct ablation experiments to study the contribution of self-supervised pre-training and attention mechanism to performance improvement (Fig. 4). Supplementary Fig. S7 shows the CFAGO model that removed multi-head attention mechanism. We only use the m-AUPR, M-AUPR, and Fmax measures, as F1 and ACC measures depend on special thresholds.

We observe that the performance drops significantly if not applying self-supervised pre-training. The reason is that the CFAGO model contains a huge amount of parameters. As there are only several thousands of training proteins, the CFAGO model is over-fitted without applying pre-training. For the multi-head attention mechanism, the performance of CFAGO drops clearly without it, except on Fmax measure of mouse MFO. The reason is that there are only 1185 mouse MFO training proteins, therefore adding the attention mechanism makes the CFAGO model overfitted, leading the performance drops down. Overall, these results also demonstrate the feature cross-fusion via multi-head attention mechanism has obvious advantages compared with concatenation of features.

### 3.5 Contribution of different features

**Figure 5:**
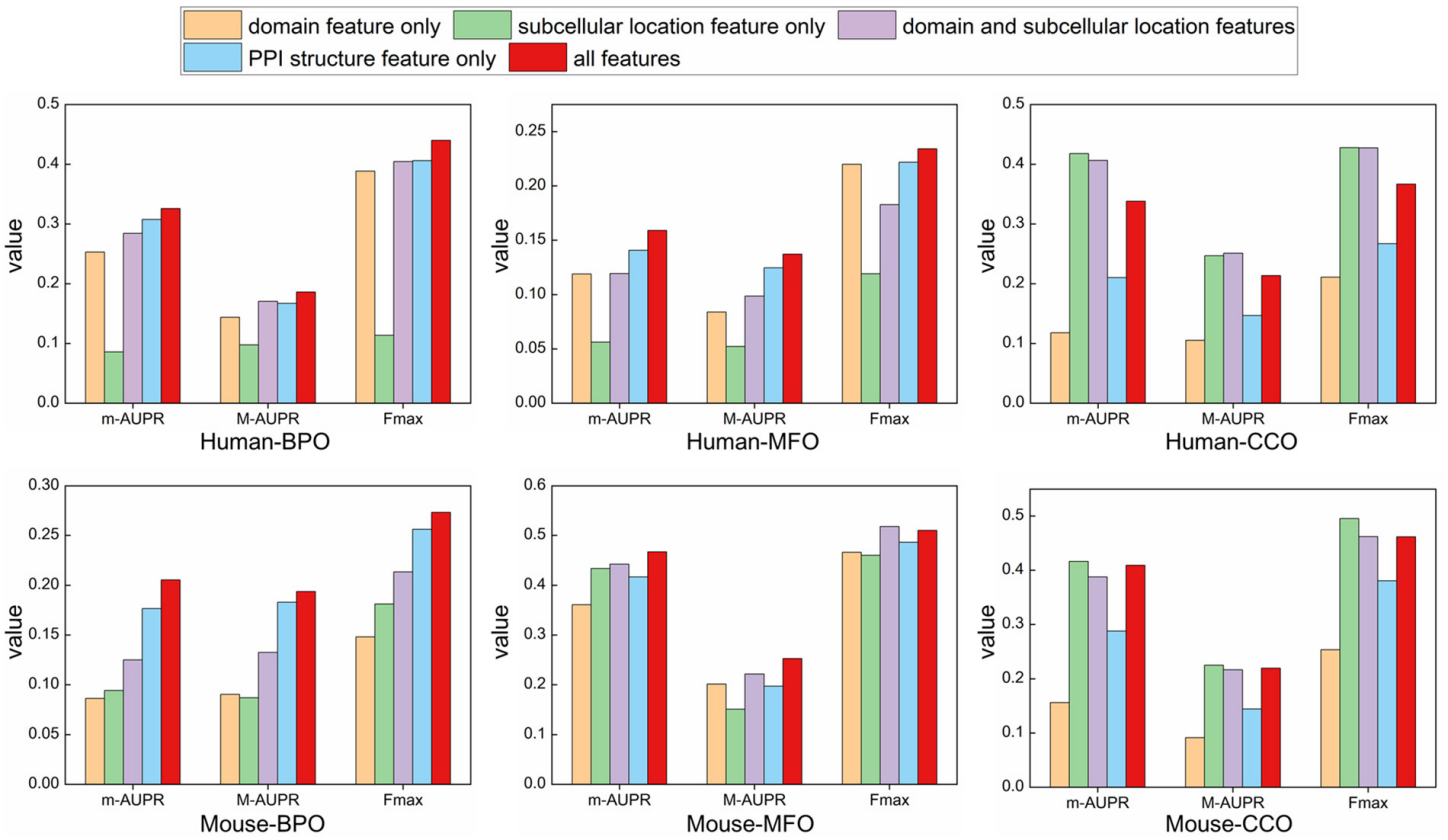
Performance comparison of different feature combinations. The meaning of different color is shown on top of the graph. m-AUPR, M-AUPR, and Fmax are performance measurements.

Here, we analyze the contribution of different features for the three GO aspects. We test five feature conditions: protein domain feature only, protein subcellular location feature only, concatenation of protein domain and subcellular location features, PPI network structure feature only, and combination of all features. Figure 5 shows the results. For BPO task, combining all of the features shows the best performance, and the PPI network structure feature contributes the most for improving prediction performance. For MFO task, combining all of the features shows the best performance, except on Fmax measure of mouse MFO. The PPI network structure feature contributes the most on human, while the combination of protein domain and subcellular location feature contributes the most on mouse. For CCO task, using the protein subcellular location feature only shows the best performance. By combining protein domain feature, the performance drops a bit, by combining PPI network structure feature, the performance drops significantly.

The reduced performance in terms of Fmax on mouse MFO is caused by the noise in protein attribute data, and insufficient number of training proteins. Protein domains came from the pfam (Mistry et al. 2021) database that contains unverified domains (Mistry et al. 2021), and the subcellular location data came from the UniProt database (UniProt 2021) that contains unreviewed records (MacDougall et al. 2020). Therefore the protein domain and subcellular location features contain noise. The reduced performance on CCO is caused by the noise in both protein attribute data and PPI data. The PPI data are produced by high-throughput techniques which contain inherent bias noise or predicted computationally (Szklarczyk et al. 2021). Besides, in the STRING database, the ratio of PPIs derived from ‘textmining’ in human dataset is 27.75%, and the ratio in mouse dataset is 19.28%. Since this kind of PPIs is predicted from scientific literature, they likely connect proteins from different subcellular locations (Szklarczyk et al. 2021). Therefore, the PPIs derived from ‘textmining’ became noise for CCO prediction.

## 4 Conclusion

In this study, we propose CFAGO, an attention mechanism-based neural network model, for protein function prediction. It cross-fuses the information from multiple sources of single species using an attention mechanism to learn more effective protein representations. Specifically, CFAGO is the first pre-trained via an encoder–decoder architecture to learn the universal protein representations and then fine-tuned to further improve protein function prediction. We show that CFAGO outperforms state-of-the-art single-species network-based protein function prediction methods in both human and mouse organisms. CFAGO would be an effective tool for understanding disease mechanisms or finding drug targets.

Several studies (Barot et al. 2021; You et al. 2021) have shown that integrating PPI networks of multiple species can further improve the accuracy of protein function prediction. In future work, we will try more types of protein attributes such as sequence features, and explore effective ways that can use full homology information to integrate PPI networks of multi-species for protein function prediction.

## Supplementary Material

btad123_Supplementary_Data
