# Supplementary material for: CFAGO: cross-fusion of network and attributes based on attention mechanism for protein function prediction
Source: Bioinformatics. 2023 Mar 8;39(3):btad123. doi: 10.1093/bioinformatics/btad123 (PMC10032634; doi:10.1093/bioinformatics/btad123)
Supplement: btad123_Supplementary_Data [file btad123_supplementary_data.docx]

**Supplementary materials**


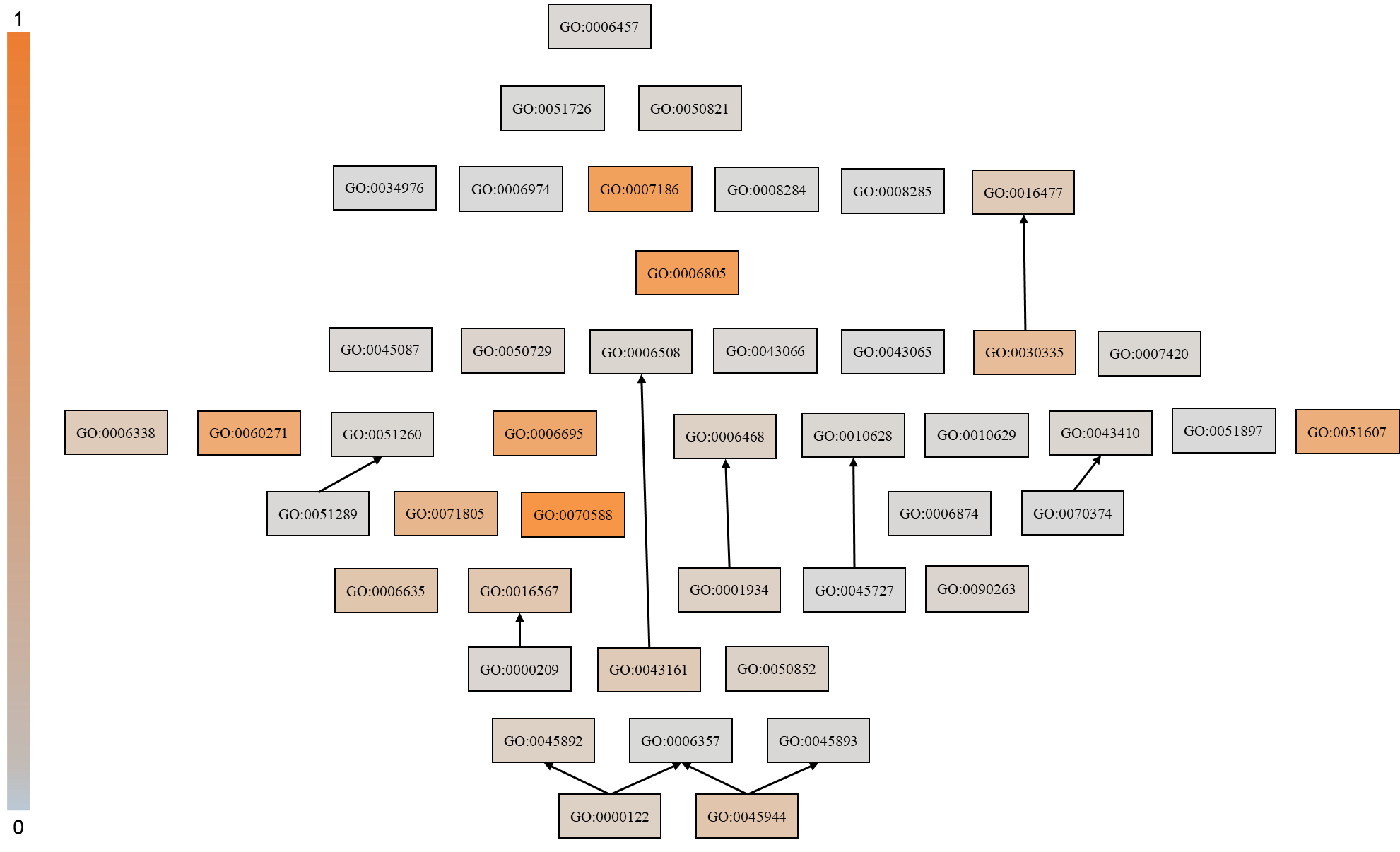


**Figure S1**. AUPR performance of CFAGO in human BPO individual terms. The GO terms are placed in relative position according to their visualization results output by the [AmiGO 2](http://amigo.geneontology.org/visualize?mode=client_amigo) tool. The arrow means there is a relation path from source term to target term, where a source term is a ‘child’ to the target ‘parent’ term, with ‘child’ terms being more specialized than their ‘parent’ terms.


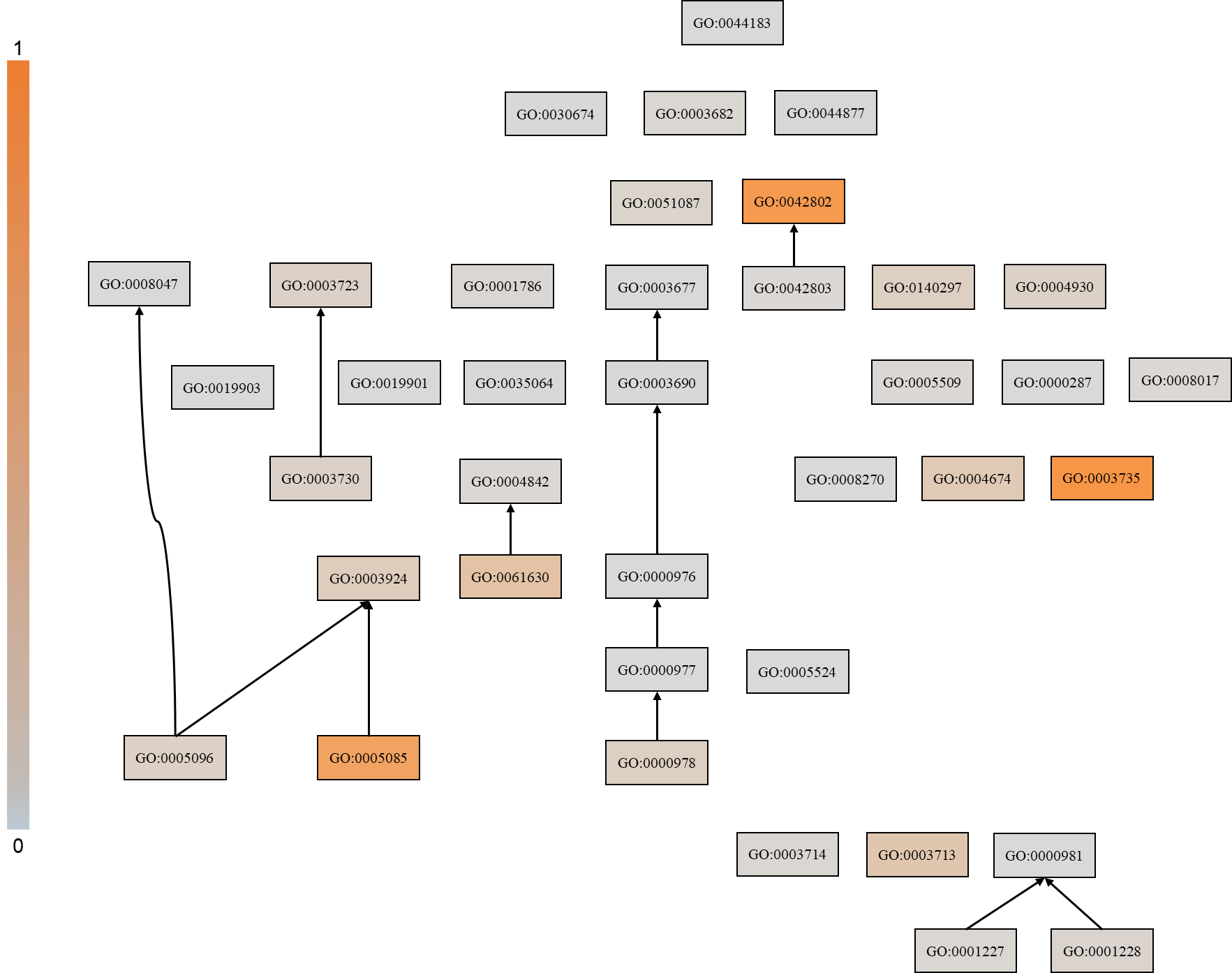


**Figure S2**. AUPR performance of CFAGO in human MFO individual terms. The GO terms are placed in relative position according to their visualization results output by the [AmiGO 2](http://amigo.geneontology.org/visualize?mode=client_amigo) tool. The arrow means there is a relation path from source term to target term, where a source term is a ‘child’ to the target ‘parent’ term, with ‘child’ terms being more specialized than their ‘parent’ terms.


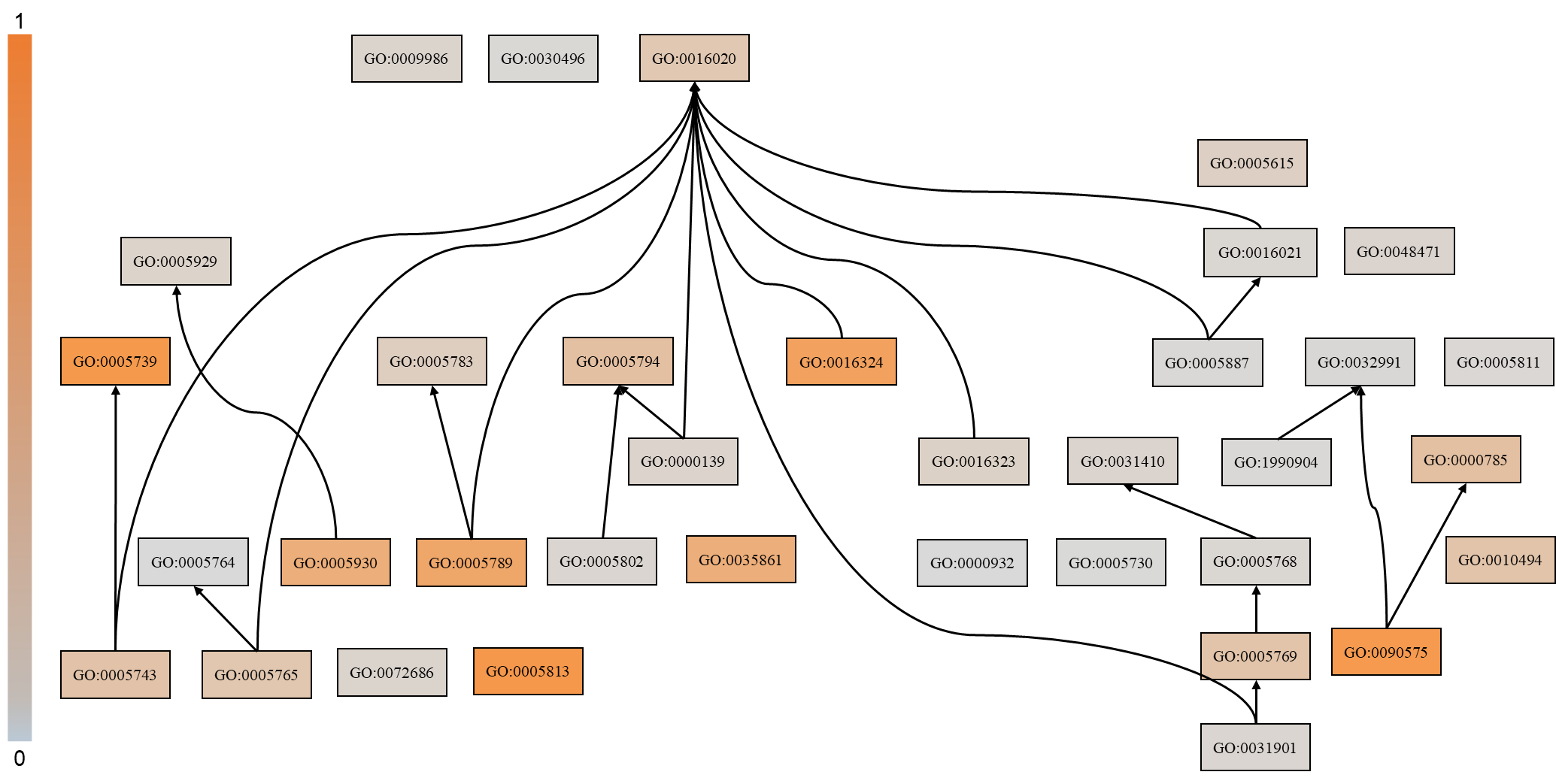


**Figure S3**. AUPR performance of CFAGO in human CCO individual terms. The GO terms are placed in relative position according to their visualization results output by the [AmiGO 2](http://amigo.geneontology.org/visualize?mode=client_amigo) tool. The arrow means there is a relation path from source term to target term, where a source term is a ‘child’ to the target ‘parent’ term, with ‘child’ terms being more specialized than their ‘parent’ terms.


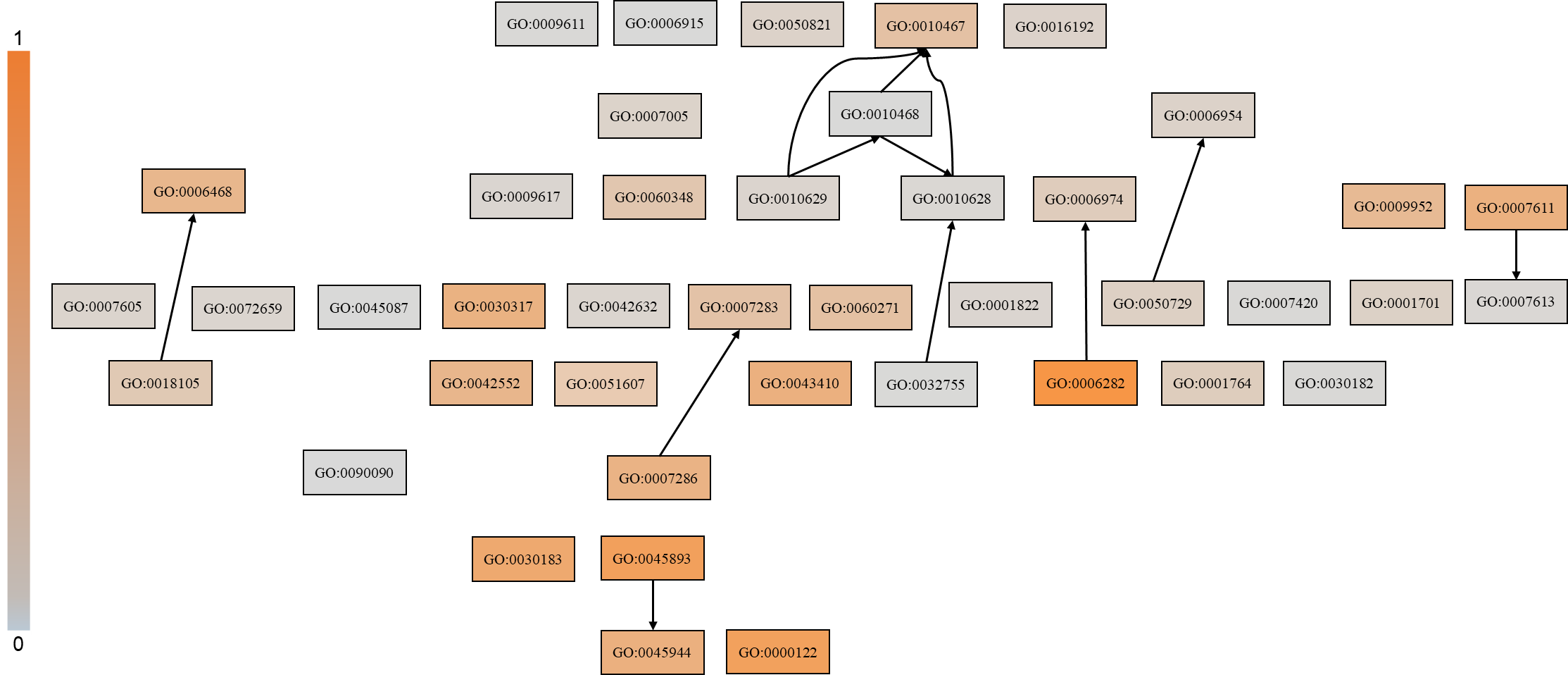


**Figure S4**. AUPR performance of CFAGO in mouse BPO individual terms. The GO terms are placed in relative position according to their visualization results output by the [AmiGO 2](http://amigo.geneontology.org/visualize?mode=client_amigo) tool. The arrow means there is a relation path from source term to target term, where a source term is a ‘child’ to the target ‘parent’ term, with ‘child’ terms being more specialized than their ‘parent’ terms.


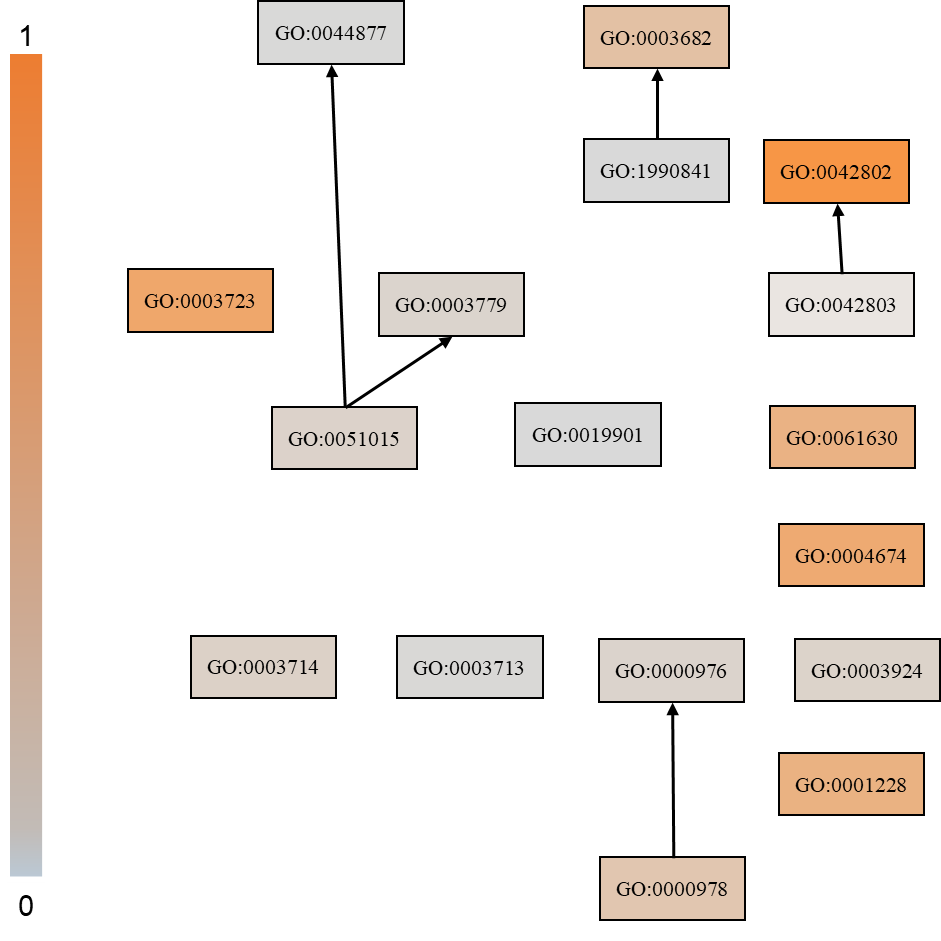


**Figure S5**. AUPR performance of CFAGO in mouse MFO individual terms. The GO terms are placed in relative position according to their visualization results output by the [AmiGO 2](http://amigo.geneontology.org/visualize?mode=client_amigo) tool. The arrow means there is a relation path from source term to target term, where a source term is a ‘child’ to the target ‘parent’ term, with ‘child’ terms being more specialized than their ‘parent’ terms.


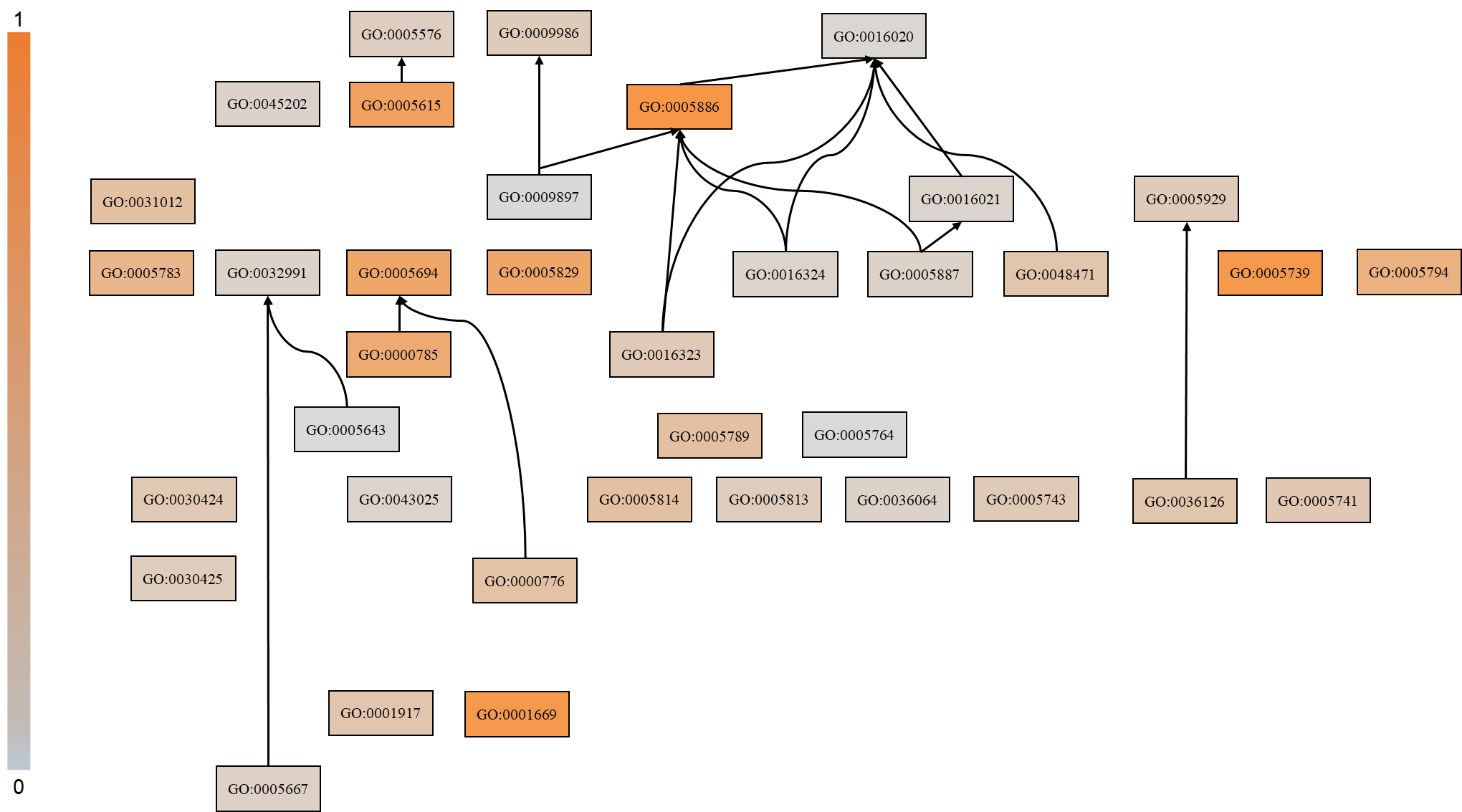


**Figure S6**. AUPR performance of CFAGO in mouse CCO individual terms. The GO terms are placed in relative position according to their visualization results output by the [AmiGO 2](http://amigo.geneontology.org/visualize?mode=client_amigo) tool. The arrow means there is a relation path from source term to target term, where a source term is a ‘child’ to the target ‘parent’ term, with ‘child’ terms being more specialized than their ‘parent’ terms.


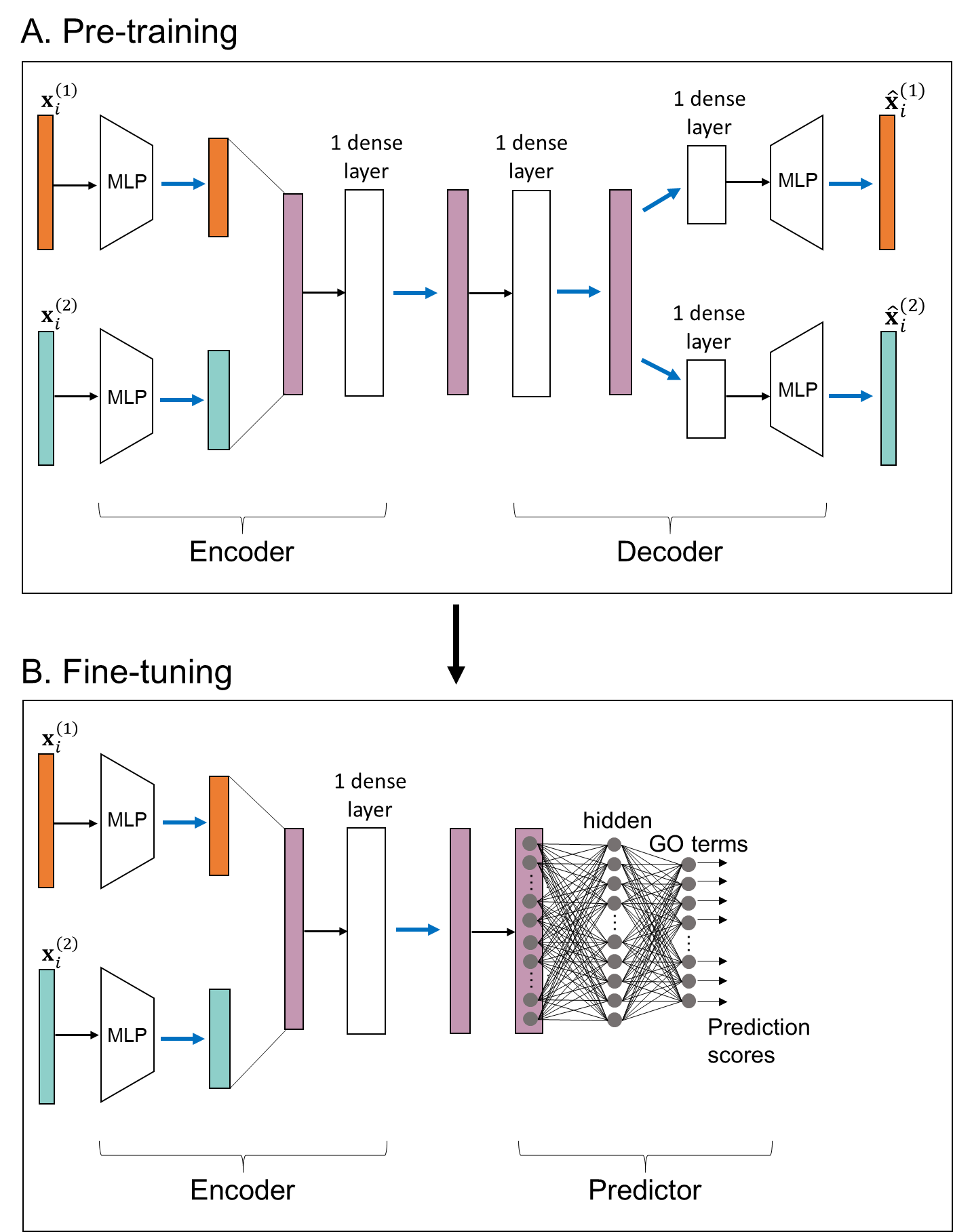


**Figure S7**. The architecture of CFAGO model without attention mechanism. The protein information from multiple sources is cross-fused by 1 dense layer, instead of multi-head attention layers.
